# A retrospective analysis of the incidence of postoperative delirium and the importance of database selection for its definition

**DOI:** 10.1186/s12888-023-04576-4

**Published:** 2023-02-06

**Authors:** Qinfeng Yang, Jinlang Fu, Xin Pan, Danping Shi, Kunlian Li, Min Sun, Jie Ding, Zhanjun Shi, Jian Wang

**Affiliations:** 1 Division of Orthopaedic Surgery, Department of Orthopaedics, Nanfang Hospital, Southern Medical University, 1838 Guangzhou Avenue, Guangzhou, 510515 Guangdong China; 2 Department of Neurosurgery, Nanfang Hospital, Southern Medical University, Guangzhou, 510515 Guangdong China; 3 Beijing Goodwill Hessian Health Technology Co., Ltd, Gehua Tower, No.1 Qinglong Hutong, Dongcheng District, Beijing, 100007 China; 4 Huiqiao Medical Center, Nanfang Hospital, Southern Medical University, 1838 Guangzhou Avenue, Guangzhou, 510515 Guangdong China

**Keywords:** Postoperative delirium, Shoulder arthroplasty, Nationwide inpatient sample

## Abstract

**Background:**

Postoperative delirium (POD) is a common complication after major surgery, resulting in various adverse reactions. However, incidence and risk factors associated with POD after shoulder arthroplasty (SA) have not been well studied using a large-scale national database.

**Methods:**

A retrospective database analysis was performed based on the Nationwide Inpatient Sample (NIS) from 2005 to 2014, the largest fully paid hospital care database in the United States. Patients undergoing SA were included. The patient’s demographics, comorbidities, length of stay (LOS), total costs, type of insurance, type of hospital, in-hospital mortality, and medical and surgical perioperative complications were assessed.

**Results:**

A total of 115,147 SA patients were obtained from the NIS database. The general incidence of delirium after SA was 0.89%, peaking in 2010. Patients with delirium after SA had more comorbidities, prolonged LOS, increased hospitalization costs, and higher in-hospital mortality (*P* < 0.0001). These patients were associated with medical complications during hospitalization, including acute renal failure, acute myocardial infarction, pneumonia, pulmonary embolism, stroke, urinary tract infection, sepsis, continuous invasive mechanical ventilation, blood transfusion, and overall perioperative complications. Risk factors associated with POD include advanced age, neurological disease, depression, psychosis, fluid and electrolyte disturbances, and renal failure. Protective factors include elective hospital admissions and private insurance.

**Conclusion:**

The incidence of delirium after SA is relatively low. Delirium after SA was associated with increased comorbidities, LOS, overall costs, Medicare coverage, mortality, and perioperative complications. Studying risk factors for POD can help ensure appropriate management and mitigate its consequences. Meanwhile, we found some limitations of this type of research and the need to establish a country-based POD database, including further clearly defining the diagnostic criteria for POD, investigating risk factors and continuing to collect data after discharge (30 days or more), so as to further improve patient preoperative optimization and management.

## Background

In recent years, studies have demonstrated that shoulder arthroplasty (SA) is a reliable option for relieving shoulder pain and improving function in patients for whom nonoperative management of glenohumeral arthritis has failed [1–4]. In the United States, SAs are reported to be growing at a comparable rate or even higher than total hip and knee procedures [5, 6]. In addition, predictive studies on the National Inpatient Sample (NIS) found that the demand for SA in the United States is probably going to keep rising [5–7]. According to the NIS analysis in the United States, the linear model predicts that the volume of SAs will reach 174,810 procedures by 2025, while the Poisson model predicts that it will reach 350,558 procedures by 2025 [8]. Nevertheless, quite a few postoperative patients suffer from postoperative complications.

Delirium is a clinical syndrome characterized by disturbances in consciousness, cognitive function, or perception. It is thought to be caused by the brain’s maladaptation to the surgical stress [9, 10]. Postoperative delirium (POD) is a common complication in geriatric patients after major surgery [11, 12]. Notably, POD is one of the common complications after total joint arthroplasty (TJA), which imposes a heavy burden on individuals and society in economic and other aspects [13]. It is strongly associated with increased mortality and morbidity, longer hospital stays, and worse surgical outcomes [14–16]. Approximately 2.4 million hospitalized elderly patients suffer from delirium at a cost of between $143 billion and $152 billion per year [17]. Furthermore, POD adversely affects patients, their family members, and healthcare workers as it is associated with higher mortality, progressive injury, long-term cognitive impairment, and other complications [18–25].

Herein, it is important for preoperative identification of patients at high risk for POD to optimize postoperative outcomes and prevent complications [13, 26–28]. Several risk factors associated with POD have been reported in the literature, with advanced age being one of the most commonly recognized risk factors [13, 26–33]. Meanwhile, other risk factors have also been identified, including dementia, depression, cognitive impairment, postoperative electrolyte disturbances, and a history of Parkinson’s disease [13, 27–33]. Besides, our previous study found that there are some risk factors for POD in patients undergoing total hip arthroplasty or total knee arthroplasty [34, 35]. These risk factors include advanced age, alcohol or drug abuse, depression, neurological disorders, psychosis, fluid and electrolyte disturbances, diabetes, weight loss, deficiency anemia, coagulation disorders, hypertension, congestive heart failure, valvular disease, Pulmonary circulation disorder, peripheral vascular disease, and renal failure. However, incidence and risk factors associated with postoperative delirium after SA have not been well studied using a large-scale national database [36].

Therefore, the purpose of this study was to explore the incidence of delirium and associated risk factors of delirium after SA based on a national database, assuming a relatively low incidence of POD and multiple risk factors, to highlight patients who may require a preoperative optimization group. Delirium incidence, patient demographics, number of comorbidities, length of stay (LOS), type of hospital, total charge and in-hospital mortality were assessed primarily. However, this retrospective analysis is limited by the lack of information on dementia history, type of anesthesia, perioperative medication, and sensory disturbances in this database. Then, complications and risk factors associated with POD were further evaluated by analyzing preoperative comorbidities, and postoperative major and minor perioperative complications in patients with SA.

## Methods

### Data source

The NIS database, part of the Agency for Healthcare Research and Quality’s Healthcare Cost and Utilization Program, was the data source for the study. The NIS represents the largest database of fully paid hospitalizations in the United States. NIS collects stratified samples from more than 1000 hospitals, accounting for approximately 20% of annual hospital admissions in the United States [37]. Information was obtained from the database, including patient demographics, service levels, total hospitalization costs, diagnoses, and procedural codes in the International Classification of Diseases (Ninth Edition) Clinical Modification (ICD-9-CM).

### Data collection

Data were from the NIS database (2005 to 2014). Patients were identified according to the ICD-9-CM SA procedure codes (81.80/81.88/81.81). According to the ICD-9-CM diagnostic code, patients with delirium were diagnosed and selected including transient, acute, and subacute delirium (293, 293.0, 293.1, 293.8, 293.9, 293.81–84, 293.89), drug-induced delirium (292.81) [18], and altered mental state (780.97). Patients younger than 18 years, with osteomyelitis or pathological fractures were excluded.

Recruits were divided into two groups based on the occurrence of POD. Patient demographics, including age, gender, and ethnicity, were assessed. Outcome measures such as mode of admission, preoperative comorbidities, LOS, the total hospital stay cost, type of insurance, and in-hospital mortality were analyzed. The ICD-9-CM diagnostic code was applied to acquire preoperative comorbidities that may be independently associated with POD, as well as medical and surgical perioperative complications before discharge. Preoperative comorbidities included Parkinson disease, dementia, anxiety, epilepsy, frailty, sleep apnea, and smoking. Perioperative medical complications were defined as acute renal failure, acute myocardial infarction, pneumonia, pulmonary embolism, stroke, urinary tract infection, deep vein thrombosis, sepsis, postoperative shock, continuous invasive mechanical ventilation, and transfusion of blood. Perioperative medical complications included periprosthetic joint infection, dislocation of a prosthetic joint, wound dehiscence/non-healing, hemorrhage/seroma/hematoma, irrigation and debridement, and injury to the peripheral nerve of the upper limb [37]. According to the database of the NIS, 29 variables of comorbidities include AIDS, alcohol abuse, deficiency anemia, rheumatoid arthritis/collagen vascular diseases, chronic blood loss anemia, congestive heart failure, chronic pulmonary disease, coagulopathy, depression, diabetes (uncomplicated), diabetes (with chronic complications), drug abuse, hypertension, hypothyroidism, liver disease, lymphoma, fluid and electrolyte disorders, metastatic cancer, neurological disorders, obesity, paralysis, peripheral vascular disorders, psychoses, pulmonary circulation disorders, renal failure, solid tumor without metastasis, peptic ulcer disease, valvular disease, and weight loss.

### Data analysis

Statistical analysis was performed with statistical software R version 3.5.3. Significant differences between the two groups were determined by the Wilcoxon rank test (continuous data) and the chi-square test (categorical data). Univariate and multivariate logistic regression models were established to assess the association of delirium with medical and surgical perioperative complications. To determine the independent risk factors for POD and their association with other medical complications and surgical complications, binary logistic regression analysis was performed using stepwise regression. All variables, NIS-provided demographics, hospital characteristics, and preoperative comorbidities, were included in the regression analysis (Table 1). Since other NIS studies used large sample sizes, the statistical significance of the alpha level was determined by *P* ≤ 0.001 [18, 38].

**Table 1:** Variables entered into the binary logistic regression analysis

Variables Categories	Specific Variables
Patient demographics	Age (≤74 years and ≥ 75 years), sex (male and female), race (White, Black, Hispanic, Asian or Pacific Islander, Native American and Other)
Hospital characteristics	Type of admission (non-elective, elective), bed size of hospital (small, medium, large), teaching status of hospital (nonteaching, teaching), location of hospital (rural, urban), type of insurance (medicare, medicaid, private insurance, self-pay, no charge, other), location of the hospital (northeast, Midwest or north central, south, west)
Comorbidities	AIDS, alcohol abuse, deficiency anemia, rheumatoid diseases, chronic blood loss anemia, congestive heart failure, chronic pulmonary disease, coagulopathy, depression, diabetes (uncomplicated), diabetes (with chronic complications), drug abuse, hypertension, hypothyroidism, liver disease, lymphoma, fluid and electrolyte disorders, metastatic cancer, neurological disorders, obesity, paralysis, peripheral vascular disorders, psychoses, pulmonary circulation disorders, renal failure, solid tumor without metastasis, peptic ulcer disease, valvular disease and weight loss

## Results

### Incidence of postoperative delirium in patients undergoing SA

**Fig. 1:**
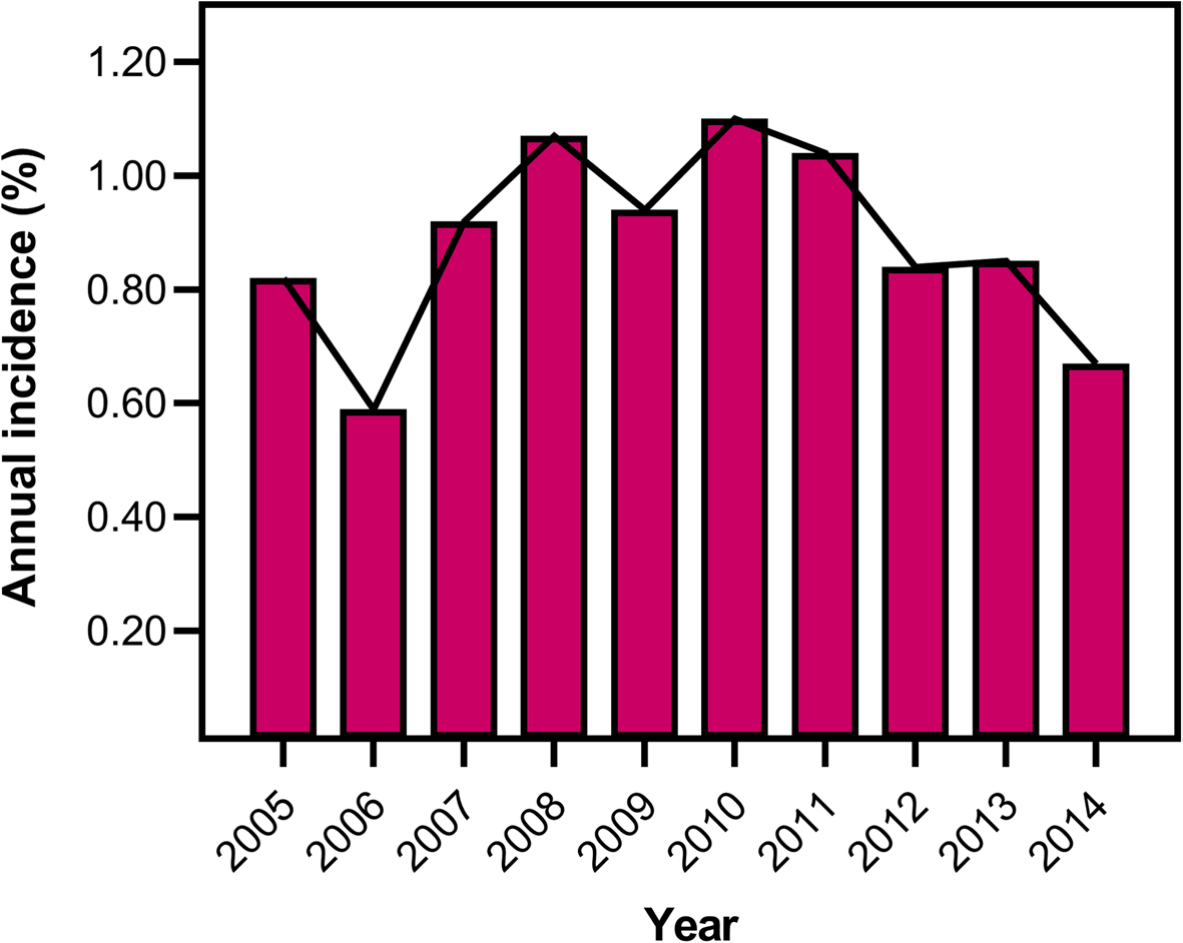
Annual Incidence of Postoperative Delirium in Patients Undergoing Shoulder Arthroplasty

From 2005 to 2014, a total of 115,147 SAs were identified in the NIS database. Overall, POD was observed in 1020 patients with an incidence of 0.89% (Table 2). The study found that the incidence of POD generally increased from 2005 (0.82%) to 2010 (1.10%) (Fig. 1), while the incidence of POD decreased annually from 2010 (1.10%) to 2014 (0.67%) (Fig. 1).

**Table 2:** Patient characteristics and outcomes of delirium after SA (2005–2014)

Parameter	No delirium	Delirium	***P***
Total (n = count)	114,127	1020	
Total incidence	0.89%		
Age (median, years)	70 (62–77)	77 (72–83)	< 0.0001
Age group (%)			
18–44	2.31%	0.49%	< 0.0001
45–64	28.68%	9.31%	
65–74	35.95%	27.06%	
≥ 75	33.05%	63.14%	
Sex (female%)	58.8%	64.51%	0.0003
Race (%)			
White	89.2%	90.97%	0.6642
Black	4.11%	3.4%	
Hispanic	3.78%	3.17%	
Asian or Pacific Islander	0.61%	0.7%	
Native American	0.39%	0.35%	
Other	1.92%	1.41%	
Elective admission (%)	88.22%	69.87%	< 0.0001
Number of Comorbidity (median)	2 (1–3)	3 (2–4)	< 0.0001
LOS (median, d)	2 (1–3)	4 (3–7)	< 0.0001
Total charges (median, $)	45,289 (32,176-64,484)	58,274 (40,603-87,813)	< 0.0001
Type of insure (%)			
Medicare	67.18%	85.46%	
Medicaid	2.59%	1.28%	
Private insurance	25.47%	10.81%	
Self-pay	0.64%	0.69%	
No charge	0.09%	0%	
Other	4.03%	1.77%	
Bed size of hospital (%)			
Small	18.44%	16.75%	0.3670
Medium	24.89%	25.02%	
Large	56.67%	58.23%	
Type of hospital (teaching %)	48.26%	49.95%	0.2979
Location of hospital (urban %)	89.46%	88.77%	0.5049
Region of hospital (%)			
Northeast	13.99%	15.69%	0.3277
Midwest or North Central	27.4%	28.14%	
South	36.79%	35.69%	
West	21.83%	20.49%	
In-hospital mortality (%)	0.12%	1.18%	< 0.0001

*SA* Shoulder arthroplasty, *LOS* length of stay

### Patient demographics and hospital characteristics between the two surgical groups

**Fig. 2:**
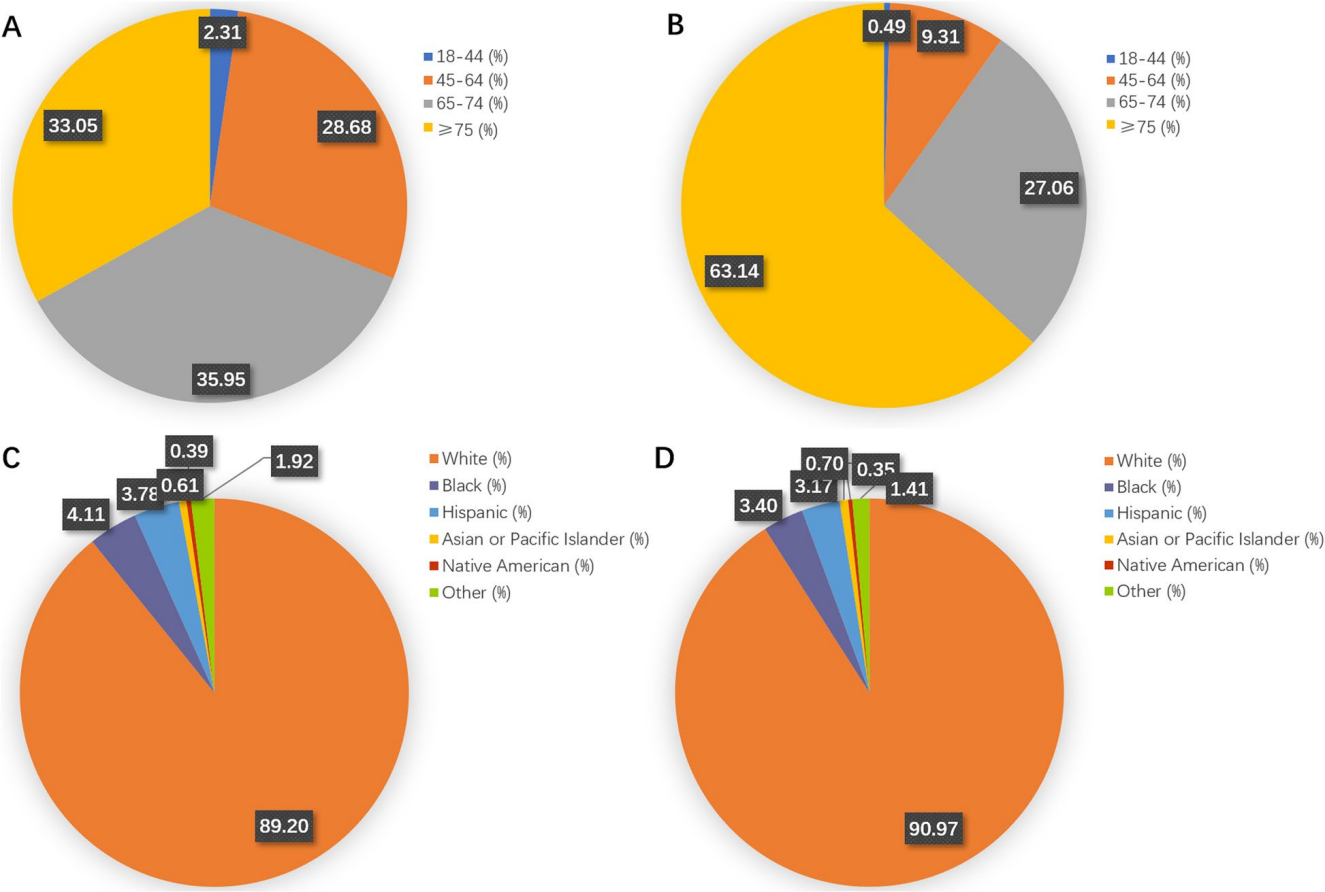
Patient demographics and hospital characteristics between the two surgical groups. **A** Age distribution analysis of non-delirium patients. **B** Analysis of age distribution of patients with delirium. **C** Racial distribution analysis of non-delirium patients. **D** Racial distribution analysis of delirium patients

**Fig. 3:**
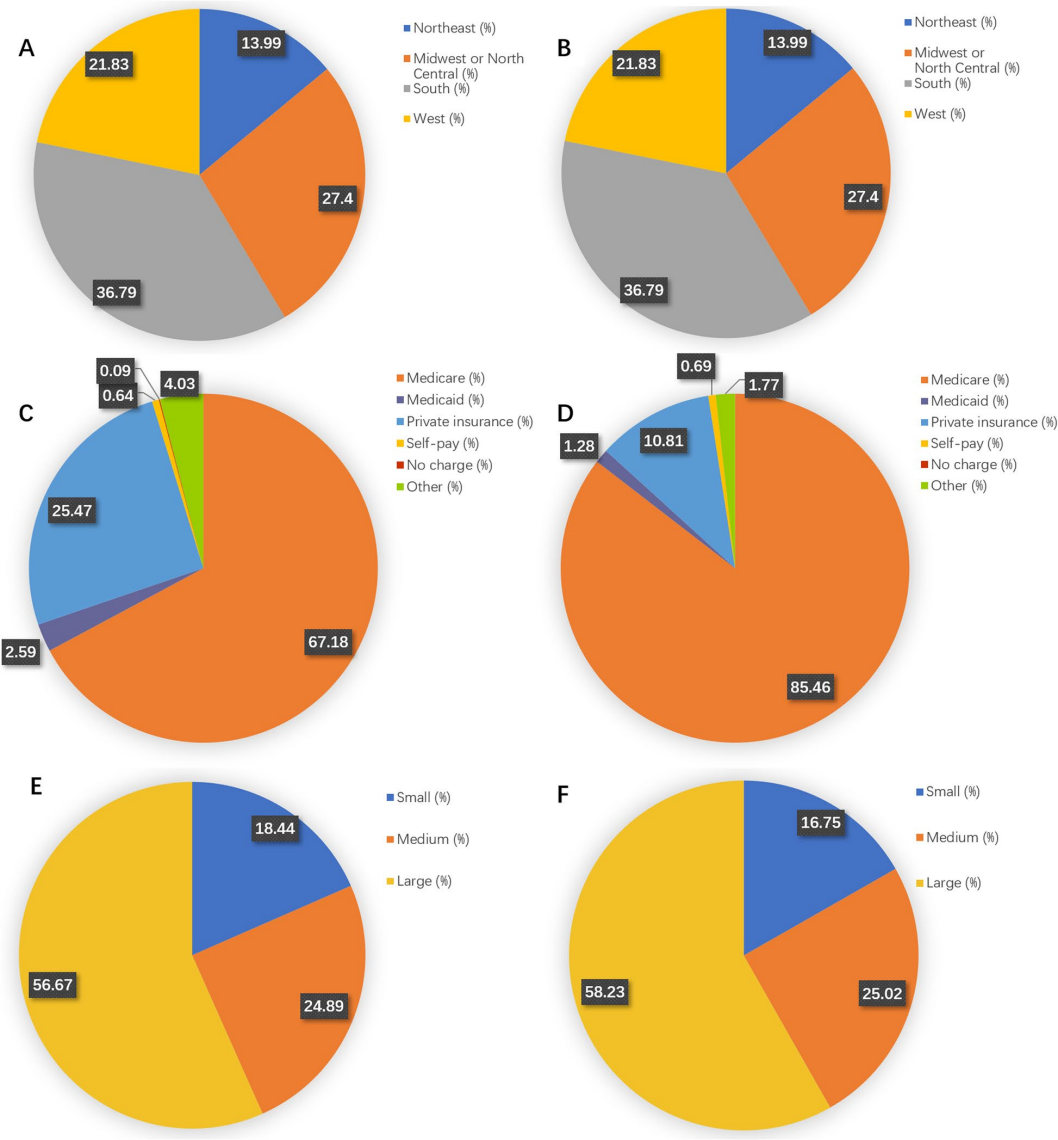
Incidence of Postoperative Complications Related to Postoperative Delirium. **A** Analysis of hospital regional distribution of non-delirium patients. **B** Analysis of hospital regional distribution of delirium patients. **C** Analysis of Insurance Types for Non-delirium Patients. **D** Analysis of Insurance Types for Patients with Delirium. **E** Analysis of the number of hospital beds for non-delirium patients. **F** Analysis of the number of hospital beds for patients with delirium

The incidence of POD was significantly different between men and women, with a higher proportion of women exhibiting delirium than men (*P* < 0.0001) (Table 2). Patients with POD (72–83 years old) were significantly older than those without POD (62–77 years old) (*P* < 0.0001). Consistently, there was a significant difference in the age distribution between the two groups (Fig. 2A & B), with a significantly higher incidence in patients over 75 years of age (*P* < 0.0001) (Table 2). However, there were no statistical differences in variables such as race, insurance type, bed size of hospital, region of hospital, etc. (Table 2 and Figs. 2C & D and 3).

### Adverse effects of postoperative delirium after SA

Patients with POD exhibited a significantly higher number of comorbidity (3 vs 2, *P* < 0.0001), as previously described. Not surprisingly, in-hospital mortality increased from 0.12 to 1.18% with POD (*P* < 0.0001) (Table 2). Patients with delirium had a longer median LOS than patients without delirium (4 days vs. 2 days; *P* < 0.0001) (Table 2). Therefore, POD increases medical costs. In the case of POD, the total charges of hospitalization increased by $12,985 in the median ($58,274 vs. $45,289, *P* < 0.0001) (Table 2). At the same time, Patients with delirium are less likely to have elective admissions (69.87% vs. 88.22%; *P* < 0.0001) (Table 2).

### Association between postoperative delirium and other preoperative comorbidities

**Fig. 4:**
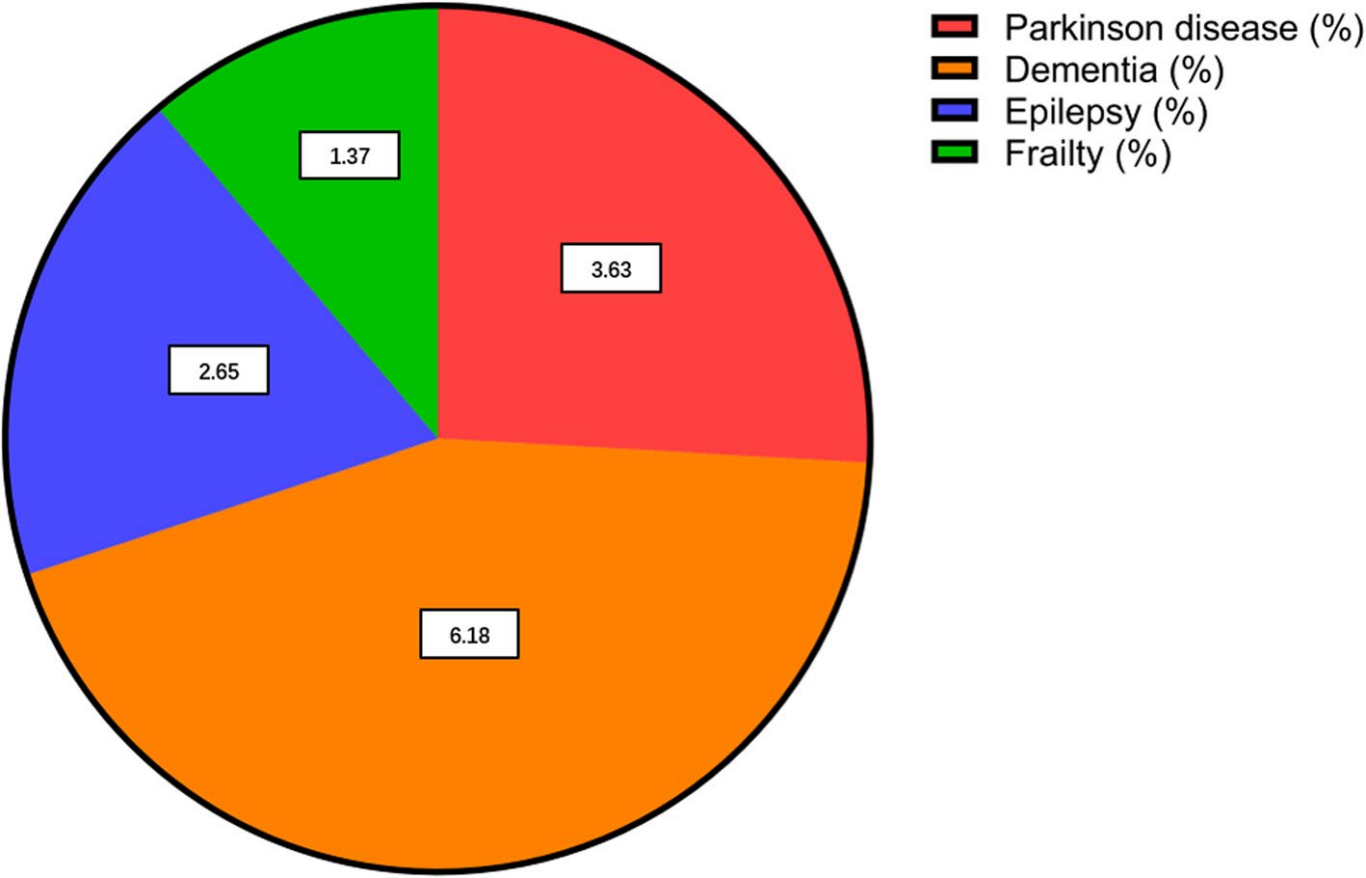
Incidence of postoperative delirium-related preoperative complications

POD patients were mostly aging patients, and patients with preoperative comorbidities such as Parkinson’s disease (3.63%), dementia (6.18%), epilepsy (2.65%), and frailty (1.37%) were more likely to have POD (*P* < 0.0001). (Table 3 and Fig. 4). The results of Logistic regression analysis were as follows: Parkinson’s disease (odds ratio [OR] = 3.36; 95% confidence interval [CI] = 2.40–4.70), dementia (OR = 4.72; CI = 3.62–6.15), frailty (OR = 4.53; CI = 2.90–7.02).

**Table 3:** Association between postoperative delirium and other preoperative comorbidities

Complication	Univariate Analysis			Multivariate Logistic Regression		
	No delirium (n,%)	Delirium (n,%)	***P***	OR	95% CI	***P***
**Preoperative complications**						
Parkinson disease	1141 (1.00%)	37 (3.63%)	< 0.0001	3.36	2.40–4.70	< 0.0001
Dementia	1378 (1.21%)	63 (6.18%)	< 0.0001	4.72	3.62–6.15	< 0.0001
Anxiety	7292 (6.39%)	93 (9.12%)	0.0005	1.41	1.13–1.75	0.0019
Epilepsy	1410 (1.24%)	27 (2.65%)	0.0001	2.03	1.38–2.99	0.0003
Frailty	312 (0.27%)	14 (1.37%)	< 0.0001	4.53	2.90–7.02	< 0.0001
Sleep apnea	11,547 (10.12%)	89 (8.73%)	0.1566	0.85	0.69–1.06	0.1542
Smoking	22,810 (19.99%)	186 (18.24%)	0.1759	0.90	0.76–1.05	0.1878

### Association between postoperative delirium and other postoperative complications

**Fig. 5:**
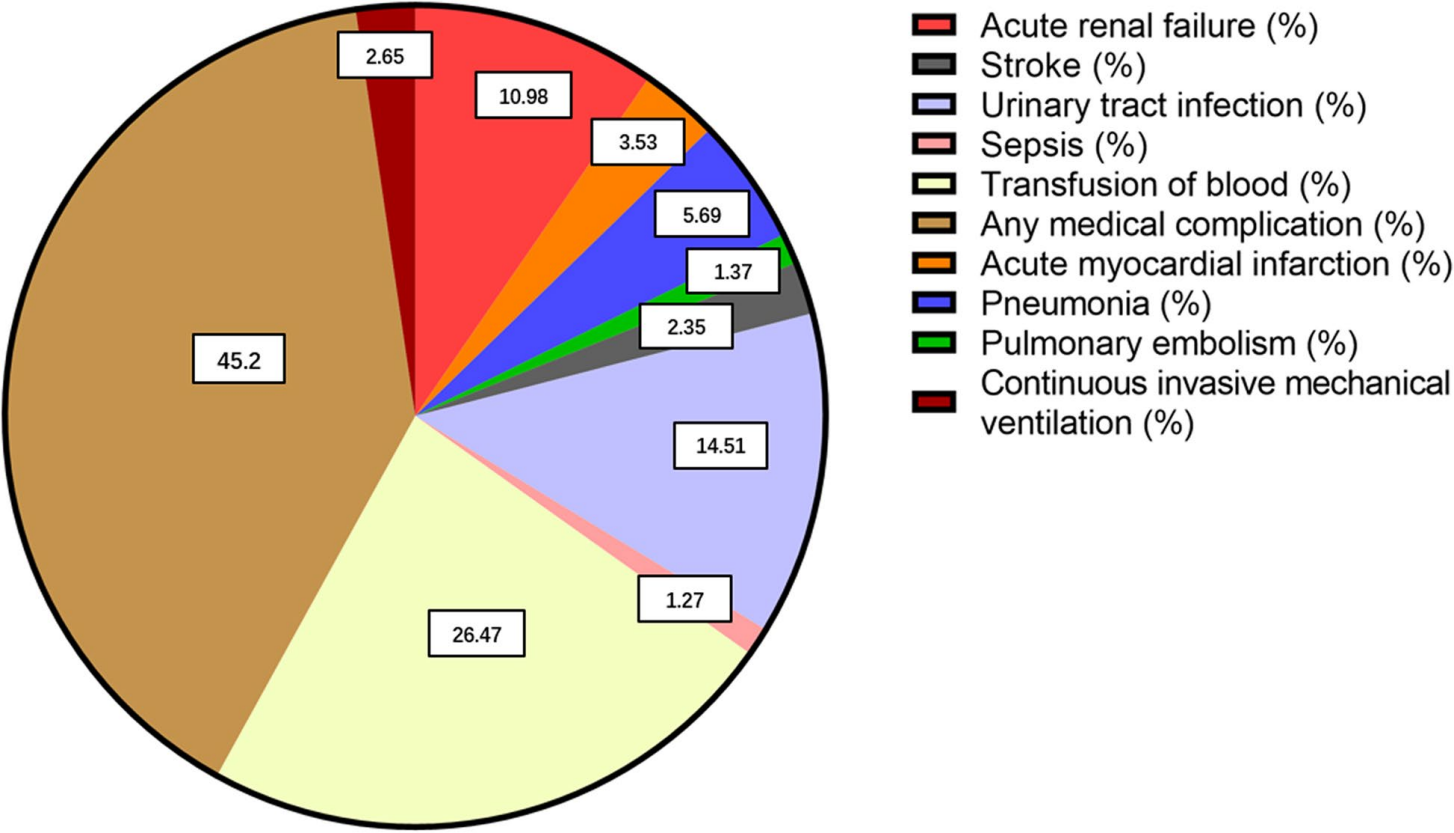
Incidence of postoperative complications related to postoperative delirium

Patients with delirium were more likely to develop acute renal failure (10.98%), acute myocardial infarction (3.53%), pneumonia (5.69%), pulmonary embolism (1.37%), stroke (2.35%), urinary tract infection (14.51%), sepsis (1.27%), continuous invasive mechanical ventilation (2.65%), blood transfusion (26.47%), and overall perioperative complications (45.2%) than patients without delirium (*P* < 0.0001). (Table 4 and Fig. 5). Multiple regression analysis found that POD was associated with acute renal failure (OR = 2.44; CI = 1.93–3.06), pneumonia (OR = 2.77; CI = 2.04–3.77) and any medical complication (OR = 2.59; CI = 2.04–3.28) (Table 4). Furthermore, in multiple analyses, delirium was not associated with any perioperative complications (Table 4).

**Table 4:** Association between postoperative delirium and other postoperative complications

Complication	Univariate Analysis			Multivariate Logistic Regression		
	No delirium	Delirium	***P***	OR	95% CI	***P***
**Medical complications**						
Acute renal failure	1888 (1.65%)	112 (10.98%)	< 0.0001	2.44	1.93–3.06	< 0.0001
Acute myocardial infarction	732 (0.64%)	36 (3.53%)	< 0.0001	1.87	1.30–2.69	0.0007
Pneumonia	776 (0.68%)	58 (5.69%)	< 0.0001	2.77	2.04–3.77	< 0.0001
Pulmonary embolism	241 (0.21%)	14 (1.37%)	< 0.0001	2.35	1.33–4.18	0.0035
Stroke	842 (0.74%)	24 (2.35%)	< 0.0001	1.39	0.90–2.14	0.1390
Urinary tract infection	3498 (3.07%)	148 (14.51%)	< 0.0001	2.01	1.62–2.51	0.0000
Deep vein thrombosis	280 (0.25%)	9 (0.88%)	0.0012	0.70	0.34–1.41	0.3160
Sepsis	179 (0.16%)	13 (1.27%)	< 0.0001	0.89	0.47–1.68	0.7272
Postoperative shock	32 (0.03%)	1 (0.1%)	0.2545	0.57	0.07–4.44	0.5912
Continuous invasive mechanical ventilation	510 (0.45%)	27 (2.65%)	< 0.0001	1.24	0.80–1.92	0.3446
Transfusion of blood	8770 (7.68%)	270 (26.47%)	< 0.0001	1.57	1.27–1.94	< 0.0001
Any medical complication^a^	14,482 (12.69%)	461 (45.2%)	< 0.0001	2.59	2.04–3.28	< 0.0001
**Surgical complications**						
Periprosthetic joint infection	227 (0.2%)	4 (0.39%)	0.1504	0.93	0.19–4.48	0.9299
Dislocation of prosthetic joint	275 (0.24%)	6 (0.59%)	0.0404	1.40	0.24–7.99	0.7083
Wound dehiscence/Non-healing	45 (0.04%)	1 (0.1%)	0.3359	1.30	0.14–12.21	0.8166
Hemorrhage/seroma/hematoma	485 (0.42%)	9 (0.88%)	0.046	0.88	0.18–4.29	0.8726
Irrigation and debridement	334 (0.29%)	5 (0.49%)	0.233	0.98	0.20–4.89	0.9840
Injury to peripheral nerve of upper limb	141 (0.12%)	2 (0.2%)	0.3618	1.03	0.13–8.46	0.9776
Any surgical complication^b^	1420 (1.24%)	25 (2.45%)	0.0009	1.05	0.21–5.18	0.9567

Any major complication^a^ or surgical complication^b^: patients with more than one complication are counted only once

### Risk factors associated with postoperative delirium after SA

Logistic regression analysis was used to investigate risk factors associated with POD (Table 5), and to identify the following indicators: advanced age (≥ 75 years, OR = 2.68; 95% CI = 2.28–3.15; *P* < 0.0001), number of comorbidity = 2 (OR = 2.45; CI = 1.56–3.86; *P* = 0.0001), number of comorbidity≥3 (OR = 3.08; CI = 1.86–5.09; *P* < 0.0001), depression (OR = 1.41; CI = 1.17–1.69, *P* = 0.0002), fluid and electrolyte disorders (OR = 2.19; CI = 1.83–2.61; *P* < 0.0001) and neurological disease (OR = 5.23; CI = 4.43–6.17). Interestingly, two protective factors were found to be associated with POD, elective admission (OR = 0.53; CI = 0.45–0.62; *P* < 0.0001) and private insurance (OR = 0.64; CI = 0.50–0.80; *P* = 0.0002).

**Table 5:** Risk factors associated with postoperative delirium after SA

Variable	Odds Ratio	95% Confidence Interval	***P***
Age ≥ 75 yr	2.68	2.28–3.15	< 0.0001
Female	0.79	0.68–0.92	0.0030
Race			
White	Ref	–	–
Black	1.04	0.70–1.54	0.8446
Hispanic	0.82	0.55–1.21	0.3182
Asian or Pacific Islander	1.12	0.49–2.56	0.7830
Native American	1.08	0.34–3.41	0.8963
Other	0.75	0.42–1.34	0.3293
Elective admission	0.53	0.45–0.62	< 0.0001
Bed size of hospital			
Small	Ref	–	–
Medium	1.00	0.81–1.24	0.9898
Large	0.96	0.79–1.16	0.6742
Teaching hospital	1.08	0.93–1.25	0.3279
Urban hospital	0.90	0.71–1.15	0.4058
Region of hospital			
Northeast	Ref	–	–
Midwest or North Central	0.81	0.65–1.02	0.0687
South	0.80	0.66–0.98	0.0307
West	0.85	0.68–1.07	0.1657
Type of insurance			
Medicare	Ref	–	–
Medicaid	0.50	0.28–0.91	0.0241
Private insurance	0.64	0.50–0.80	0.0002
Self-pay	0.66	0.26–1.65	0.3755
No charge	0.00	0.00–4.22	0.9546
Other	0.67	0.40–1.14	0.1375
Number of Comorbidity			
0	Ref	–	–
1	1.75	1.12–2.74	0.0141
2	2.45	1.56–3.86	0.0001
≥ 3	3.08	1.86–5.10	< 0.0001
AIDS	0.00	0.00–1.43	0.9665
Alcohol abuse	1.80	1.24–2.60	0.0020
Deficiency anemia	1.14	0.95–1.38	0.1549
Arthralgia	0.98	0.73–1.32	0.8964
Chronic blood loss anemia	1.03	0.61–1.73	0.9230
Congestive heart failure	1.34	1.06–1.70	0.0153
Chronic pulmonary disease	1.00	0.84–1.19	0.9912
Coagulopathy	1.21	0.84–1.74	0.2977
Depression	1.41	1.17–1.69	0.0002
Diabetes, uncomplicated	0.89	0.74–1.07	0.2182
Diabetes with chronic complications	1.69	1.21–2.38	0.0022
Drug abuse	1.91	1.10–3.32	0.0211
Hypertension	1.00	0.83–1.21	0.9776
Hypothyroidism	1.01	0.84–1.21	0.9221
Liver	0.91	0.50–1.67	0.7703
Lymphoma	1.30	0.53–3.22	0.5705
Fluid and electrolyte disorders	2.19	1.83–2.61	< 0.0001
Metastatic cancer	0.97	0.23–4.15	0.9722
Neurological disorders	5.23	4.43–6.17	< 0.0001
Obesity	0.97	0.78–1.20	0.7685
Paralysis	1.00	0.48–2.08	0.9924
Peripheral vascular disorders	0.96	0.70–1.32	0.7990
Psychoses	1.36	0.97–1.91	0.0724
Pulmonary circulation disorders	1.30	0.86–1.97	0.2103
Renal failure	1.17	0.92–1.47	0.1951
Tumor	1.04	0.49–2.23	0.9165
Ulcer	7.27	0.92–57.50	0.0600
Valvular disease	0.89	0.67–1.18	0.4130
Weight loss	1.66	1.06–2.59	0.0254

*SA* Shoulder arthroplasty, *OR* odds ratio, *CI* confidence interval

## Discussion

This present study represents the result of a large-scale health economic analysis of POD in SA. From the year 2005 to 2010, the incidence of POD ranged from 0.82–1.10%. Then, the incidence of POD decreased annually to 0.67% in 2014 (Fig. 1). Notably, this trend has not been reported in previous studies. Interestingly, our previous study of POD in patients undergoing total knee arthroplasty or total hip arthroplasty found the consistent tendency that the incidence of POD increased from 2005 to 2008 whereas decreased till to 2014 [34, 35]. While the definition of delirium did not change during this decade, the diagnosis of delirium according to the ICD-9-CM may vary by the agency [18]. One potential explanation for this observed increase in the incidence of delirium following SA before 2010, might be attributed to the growing recognition of this complication by hospital coders. Another potential explanation accounting for this trend may be that the number of SA performed was growing with aging of population, nevertheless, the lack of awareness and medical interventions, immature types of anesthesia, or even the protocols of relieving pain possibly caused a higher incidence of POD [18, 39]. Afterward, POD received increasing attention, a trend that was reversed after 2010.

We determined that the overall incidence of SA after surgery was 0.89%, which was much lower than most previous studies (5 to 14.3%). The incidence of delirium after TJA has been reported in the literature to range from 5 to 14.3% [13]. Whereas Lee et al. found an overall incidence of 2.4% after shoulder and elbow orthopedic surgery in Korean patients [40]. This depends on the investigator’s definition of delirium, the patient population, and the evaluation model utilized [41, 42]. Two possible reasons have to do with the apparent difference in the former. First, previous literature mostly observed small and selected elderly patients, leading to an overestimation of incidence. Secondly, differences in diagnostic accuracy between institutions may also contribute to differences [18, 39]. One possible reason for Lee et al. observed a higher incidence in the Asian population is that they looked at the incidence of delirium after orthopedic surgery on the shoulder and elbow.

Regarding demographic characteristics, patients with POD were significantly 7 years older than those without. Besides, in terms of age distribution, as observed in clinical practice, elderly patients take up a greater proportion of the POD group. Further, in logistic regression analysis, age over 75 years was identified as an independent risk factor for POD (Table 5). Many studies have shown that advanced age is a well-established predictor of POD [43, 44]. The results of the analysis of our data are very similar to those described above. The mechanism of neurocognitive dysfunction caused by advanced age is complex and unclear. One of the possible speculations is that advanced age is significantly associated with endothelial dysfunction and atherosclerosis, which leads to an increased risk of cerebral embolism [45]. Therefore, the possible explanation based on previous studies is that the inhibition of cerebral blood flow caused by postoperative inflammatory changes in elderly patients may be one of the influencing factors leading to postoperative neurocognitive dysfunction [46].

Patients suffered from POD had significantly higher comorbidity scores. This is reasonable because a higher number of comorbidities imply relatively poorer preoperative health and may increase postoperative complications, including delirium. POD has been reported to increase hospital stays, healthcare costs, and mortality [13, 29, 47–51]. Our study found similar results (Table 2). The median hospital stay was 2 days longer due to POD, and the total hospital cost per admission increased by $12,985. This may be the result of patients with POD failing to follow guidelines for care and recovery [18, 52]. Another explanation is that POD may be related to perioperative complications, including acute renal failure, acute myocardial infarction, pneumonia, pulmonary embolism, stroke, urinary tract infection, sepsis, continuous invasive mechanical ventilation, and blood transfusion (Table 4). These complications tended to delay discharge and prolong hospitalization [37, 53].

Additionally, patients without delirium were more likely to pay through private insurance than those patients with delirium (Table 2). However, private insurance was a protective factor for POD in logistic regression analyses (Table 5). One possibility is that private insurance tends to mean better economic conditions, which plays an important role in the development of POD. Besides, we found that patients undergoing SA via elective admission had a lower incidence of delirium (Table 2). Moreover, elective admission was also a protective factor in logistic regression analyses (Table 5). This is because most elective cases have either well healthy conditions or adequate evaluations and preparations preoperatively. As a result, in-hospital mortality was more than ten times higher in delirium patients than in unaffected patients.

Several studies of delirium after TJA suggest that prescreening, risk stratification and proper management are critical to improving outcomes [13, 28, 47–50]. Therefore, it is important to understand the relevant risk factors before surgery to prevent POD. Logistic regression was applied and the results were largely consistent with previous publications [13, 28–33, 47–50]. As expected, preoperative neurological diseases were associated with the highest odds ratio of delirium (OR = 5.23), and patients with preoperative neurological-related diseases had a significantly increased risk of POD (Table 4), such as Parkinson’s disease (OR = 3.36) or dementia (OR = 4.72). A prospective observational study in patients undergoing elective cardiac surgery indicates that frailty may increase patients’ risk of POD by 3–8 times [54]. Moreover, older age (≥75 years) was associated with a higher risk of delirium after SA (OR = 2.68). Other complications such as fluid and electrolyte disturbances (OR = 2.19) and renal failure (OR = 2.44) have also been reported as risk factors for delirium [31, 33, 47, 51]. Furthermore, to the authors’ knowledge, pneumonia (OR = 2.77) was identified for the first time as an independent risk factor for POD. Interestingly, elective admission (OR = 0.53) was found to be a protective factor (Table 5).

There are some limitations using the NIS database. First, each patient’s information was only recorded before discharge, meaning that any complications that occurred after discharge will not be included in the NIS database. This limitation may underestimate the incidence of POD, as only early medical records were analyzed. Secondly, only risk factors recorded in the NIS database can be analyzed. There are other known risk factors unable to be acquired in the NIS database, such as a history of dementia, type of anesthesia, commonly used perioperative medications (opioids, benzodiazepines, and ketamine), sedation during recovery from anesthesia, visual impairment, dysfunction, etc. [18, 30, 33, 47, 55]. Furthermore, the results obtained as a retrospective database analysis require to be clarified to determine their etiology.

## Conclusion

POD is a common complication in the elderly after SA, with an overall incidence of 0.89%. The annual incidence of POD rose from 2005 to 2010 but gradually declined from 2010 to 2014. This study identified several risk factors, including advanced age (≥ 75 years), history of neurological and psychiatric disorders, fluid and electrolyte disturbances, blood transfusions, congestive heart failure, pneumonia, and renal failure. However, elective admissions and private insurance were found to be protective factors. The incidence of delirium after SA was associated with increased LOS, increased total hospitalization costs, in-hospital mortality, and perioperative complications (acute renal failure, acute myocardial infarction, pneumonia, pulmonary embolism, stroke, urinary tract infection, sepsis, continuous invasive mechanical ventilation), but not surgical complications. Meanwhile, we found some limitations of this type of research and the need to establish a country-based POD database, including further clearly defining the diagnostic criteria for POD, investigating risk factors and continuing to collect data after discharge (30 days or more), so as to further improve patient preoperative optimization and management.

## Data Availability

This study is based on data provided by Nationwide Inpatient Sample (NIS) database, part of the Healthcare Cost and Utilization Project, Agency for Healthcare Research and Quality. The NIS database is a large publicly available full-payer inpatient care database in the United States and the direct web link to the database is https://www.hcup-us.ahrq.gov/db/nation/nis/nisdbdocumentation.jsp. Therefore, individual or grouped data cannot be shared by the authors.

## Abbreviations

SA: Shoulder Arthroplasty
TJA: Total Joint Arthroplasty
NIS: Nationwide Inpatient Sample
LOS: Length of stay
OR: Odds ratio
CI: Confidence interval
